# A Randomized, Double‐Blind, Placebo‐Controlled Pilot Trial With Open‐Label Extension of Sirona, a Hydrogel for Weight Loss

**DOI:** 10.1002/oby.70066

**Published:** 2025-10-15

**Authors:** James P. Byrne, Elanor C. Hinton, Asif Malik Humayun, Dimitri J. Pournaras, Rebecca L. Elsworth, Jeffrey M. Brunstrom, Julian P. Hamilton‐Shield, Mark Sumeray, Camilla Easter, Hutan Ashrafian

**Affiliations:** ^1^ NIHR Southampton Biomedical Research Centre University Hospital Southampton Southampton UK; ^2^ NIHR Bristol Biomedical Research Centre University Hospitals Bristol and Weston Trust and University of Bristol Bristol UK; ^3^ Oxford Medical Products Limited Witney UK; ^4^ Milton Keynes University Hospital Foundation Trust Milton Keynes UK; ^5^ Department of Bariatric and Metabolic Surgery North Bristol NHS Trust, Southmead Hospital Bristol UK

**Keywords:** hydrogel, obesity, RCT, weight management

## Abstract

**Objective:**

This study investigated the safety, tolerability, and preliminary efficacy of Sirona, a novel gastro‐retentive, dual‐network polymer for weight management.

**Methods:**

This pilot trial comprised a randomized, parallel‐group, double‐blind, placebo‐controlled (3:1 ratio), 12‐week period, with a 12‐week open‐label extension (OLE), in participants with BMI of 30–40 kg/m^2^. Primary endpoints were feasibility, tolerability, and safety; secondary endpoints included weight loss and dietary intake, tested using Hedge's *g* [95% CI] as a measure of effect size.

**Results:**

Participants received Sirona (*n* = 29/38) or Placebo (*n* = 9/38) (mean [SD] age = 40.9 [8.4]; weight = 101.7 [12.9] kg; BMI = 35.6 [3.0]; 29/38 female and 23/38 White British). Dosing was well tolerated (RCT Sirona: 95.2 [11.0]%; RCT Placebo: 97.8 [3.5]%; RCT + OLE Sirona: 93.1 [13.0]%). No serious adverse events occurred. Of the adverse events, nausea was most prominent (74.8%), mostly graded mild (79.3%) and requiring no intervention (84.4%). Percentage total body weight loss was greater for Sirona compared to Placebo after 12 weeks (3.9 [3.0]% versus 1.0 [2.1]%, *g* = 0.96 [−1.81, −0.10]). Weight loss continued in the OLE (change from baseline = 4.4 [3.8]%). Dietary intake reduced from baseline after 12 weeks of treatment (−382.5 [519.3] kcal; Placebo = 93.5 [670.3] kcal, *g* = −0.8 [−1.7, 0.0]) and after 24 weeks (−338.2 [486.7] kcal, *g* = 0.7 [0.2, 1.1]).

**Conclusions:**

Sirona was well tolerated, with mild, primarily gastrointestinal side effects. Reduced weight and dietary intake suggest Sirona is suitable as a nonpharmacological treatment for weight management.

**Trial Registration:**

ISRCTN14083641 (https://doi.org/10.1186/ISRCTN14083641)

**Study Importance**

- What is already known?
  ○ Sirona is a novel gastro‐retentive, dual‐network polymer that has been developed for weight management. Taken orally as a pill, the hydrogel swells rapidly in the stomach, where it remains before breaking down and passing naturally.
  ○ Early first‐in‐human data from a prospective, single‐arm, 8‐week study in participants with obesity (BMI 30–40) (*n* = 10) demonstrated that Sirona was well tolerated with a favorable safety profile and suppressed appetite and reduced food intake, with multiday gastric retention.
- What does this study add?
  ○ This 12‐week, randomized, parallel‐group, placebo‐controlled, double‐blind pilot trial of treatment with Sirona with a 12‐week open‐label extension was associated with reduced dietary intake and greater weight loss compared to placebo with a large effect size.
  ○ Sirona was well tolerated, with 93% of agreed doses taken over the 24‐week total trial. There were no serious adverse events. Reported adverse events were primarily gastrointestinal, graded as mild, and resolved without intervention.
- How might these results change the direction of research or the focus of clinical practice?
  ○ These findings suggest that Sirona is suitable as a safe, tolerable, easily administered, nonpharmacological treatment for weight management, promoting personalized obesity care by providing an alternative for those who cannot take obesity medications or after weight loss with medications.
  ○ Sirona will be tested further in a larger sample of people living with overweight and obesity within a pivotal trial to be conducted in the United States and United Kingdom.

## 1 Introduction

There are currently more than 2.5 billion people worldwide [1] and 64% of adults in the United Kingdom [2] living with overweight or obesity. Excessive weight is responsible for 90% of type 2 diabetes cases [3], is the second leading preventable cause of cancer, and is a major risk factor for cardiovascular disease [4, 5], osteoarthritis, and subfertility [6, 7]. Excessive weight costs UK society £98 billion per year, equating to 4% GDP [8], driving higher treatment costs and reduced productivity. Furthermore, US adults with obesity experienced 100% higher annual medical care costs compared to those with a normal weight [9].

The most clinically effective approach to weight loss currently involves surgical intervention, following which most patients will maintain successful long‐term weight loss [10]. However, only 1% of eligible patients received surgery as a treatment for obesity [11, 12]. Incretin‐based antiobesity medications (AOMs), including GLP‐1 and GLP‐1/GIP receptor agonists, have transformed obesity management by delivering substantial and sustained weight loss alongside metabolic benefits [13, 14, 15]. However, their use is constrained by high cost, gastrointestinal side effects, access issues [16], and the need for ongoing treatment to maintain benefits, as weight regain is often seen after cessation [17]. Therefore, there is an unmet need for safe, nonpharmacological, and cost‐effective alternatives.

Hydrogels have received considerable attention in the clinical domain (e.g., wound dressings, contact lenses), due to being biochemically inert. Hydrogel‐based solutions have been designed for weight management [18, 19], with two to date being introduced to the market for people living with overweight and obesity (body mass index [BMI] of 27–40 kg/m^2^); first, the GLOW trial of Gelesis100 demonstrated a placebo‐adjusted weight loss of 2% at 6 months, with 58.6% of participants in the treatment group achieving ≥ 5% total body weight loss (TBWL) at 6 months [20]. More recently, the RESET trial of the Epitomee capsule demonstrated a placebo‐adjusted weight loss of 2.0% at 6 months and 56% of the treatment group achieved ≥ 5% TBWL [21]. Oxford Medical Products (OMP) has developed Sirona, an inert, dual‐network polymer designed to expand within the stomach. Crucially, Sirona has a differing structure once swollen in the stomach leading to multiday gastric retention. Indeed, first‐in‐human data over an 8‐week treatment period demonstrated appetite suppression [22, 23], reduced food intake [24], and MRI indicated Sirona remained in the stomach for 2–5 days [25].

The objective of this randomized, double‐blind, placebo‐controlled, 12‐week pilot trial was primarily to assess the feasibility (recruitment, adherence, and retention), tolerability (% of agreed dose taken and patient experience), and safety (adverse events [AE] recording) of Sirona, with secondary outcomes relating to preliminary efficacy in terms of weight loss and dietary intake. A further 12‐week open‐label extension (OLE) was included to assess safety and preliminary efficacy over a 24‐week period for those randomized to the Sirona arm.

## 2 Methods

### 2.1 Trial Design

The study comprised two 12‐week periods (Figure S1): (1) randomized, double‐blind, parallel‐group, placebo‐controlled trial of daily dosing (RCT) and (2) 12‐week extension period in which all participants received daily dosing of Sirona in an open‐label design (OLE), either continuing with Sirona (Sirona arm) or starting to take Sirona (ex‐Placebo arm). The study was conducted at three centers in the National Health Service in the United Kingdom (Southampton, North Bristol, and Milton Keynes), and it was initiated in January 2024 and ended on December 5, 2024. The study was conducted in accordance with the Declaration of Helsinki, ISO 14155 for devices and the International Council for Harmonisation, including Good Clinical Practice. All relevant study documents were approved by the Health Research Authority (HRA) and South Central—Hampshire B Research Ethics Committee (22/SC/0081) before the study was initiated. The study was preregistered on the ISRCTN database (ISRCTN14083641). Patient and public involvement (PPI) sessions were held to ensure the study design met the needs of patients with lived experience of obesity, who also improved the patient‐facing documentation prior to approval.

### 2.2 Intervention

Sirona is taken orally as a pill with 250 mL water on a fasted stomach. The placebo pills (Zeebo Effect LLC) consisted of microcrystalline cellulose (96%), and they were the same size, color and shape and similar weight to Sirona pills. Gradual dose escalation allowed participants to find their ideal tolerated dose (from one pill every other day up to a maximum of two pills per day over 5 weeks), with clinical support according to patient tolerability. Guidance for improving tolerability (use of water, snacks, and rescue medication) was given to participants on the basis of their daily ratings on a patient‐reported outcome known as the Motion Sickness Severity Scale (MSSS [26]), which assesses nausea from 0 (*no symptoms*) to 6 (*vomiting*) (e.g., rating 0–2, advice was to drink fluids/eat a little plain food). Following the 5‐week period, participants continued with their agreed dose for the remainder of the trial.

### 2.3 Participants

The study population comprised adults, aged between 18 and 65 years, with BMI ≥ 30 to ≤ 40 kg/m^2^, and who had been screened by a bariatric psychologist and signed the informed consent form. The main exclusion criteria were: usage of oral medication (with the exception of over‐the‐counter medications, supplements, and omeprazole, which were allowed for the latter stages of the trial once appropriate testing had been completed), usage of AOMs, history of surgery or disorders of the gastrointestinal tract, and type 1 diabetes. Full details of the inclusion and exclusion criteria are included in Table S1.

### 2.4 Procedures

Prescreening, using targeted online advertisements and including provision of the participant information sheet, identified participants, who then attended a screening visit. After informed consent was provided, participants were asked to swallow a dummy pill to ensure they were able to swallow Sirona pills (slightly larger than the standard 000 pill size). Participants were screened for medical history, vital signs, height and weight (for BMI calculation), routine blood tests, urine sample (for pregnancy test), and mental state evaluation, and they underwent a gastroscopy to ensure the stomach was free from pathology before enrollment.

Once eligibility was confirmed, participants were randomized to either the Sirona or Placebo treatment arm in a 3:1 ratio overall, using the electronic data capture system to automatically assign each participant to a treatment arm according to the randomization schedule. Participants, site staff, investigators, and sponsor were blinded to treatment allocation, except for the unblinded site staff responsible for dispensing treatment. Participants had one appointment with a dietitian to discuss a healthy eating plan, using the Nutrition and Diet Resources (NDR) meal plans for a 1500‐cal diet [27], and recommendations for at least 30 min of walking or other physical activity each day. Online assessments, prior to treatment onset, were conducted to measure dietary intake and ecological momentary assessment of appetite* [28] (* indicates to be reported elsewhere). Participants then attended a baseline clinic visit to record weight and conduct baseline assessment of eating behavior traits (using the Three Factor Eating Questionnaire, TFEQ [29]*), quality of life (using the EQ‐5D‐3L [30]*), and an appetite assessment at 30‐min intervals for 2 h after dosing (baseline assessment was 250 mL water alone*).

Participants attended the clinic for their first dose within a week of the baseline assessment, then during Week 6 (after dose escalation), Week 13 (after 12‐week dosing), and Week 25/26 for the follow‐up visit after 24 weeks of treatment. Routine and metabolic blood tests, TFEQ, and EQ‐5D‐3L were repeated at the end of the RCT period (Week 13) prior to unblinding and again at follow‐up. Dietary intake assessments were conducted in Week 12 (prior to unblinding) and at follow‐up. Participant experience questionnaires were completed at the end of both the RCT and OLE.

### 2.5 Endpoints

Feasibility was measured by the total number of participants consented and enrolled, as well as the percentage of participants who adhered to the dosing schedule and were retained to completion of follow‐up measures. Tolerability was measured through (i) compliance with the dosing schedule, (ii) ratings on the participant experience questionnaire (regarding the acceptability of swallowing the pills and the dosing schedule), and (iii) ratings on the MSSS (daily during dose escalation). Safety was measured through reporting of AEs coded using the Medical Dictionary for Regulatory Activities (MedDRA), findings on gastroscopy, changes in vital signs, and clinical laboratory tests. Preliminary efficacy was assessed through %TBWL, change in weight (kg), waist circumference (cm), and BMI (kg/m^2^), and change in average daily dietary intake (in kcal, using the validated, 24‐h recall online system Intake24 [31, 32, 33]). Participants were asked to complete two weekday recalls and one weekend day recall at each time point (baseline, Week 12 prior to unblinding, and follow‐up).

### 2.6 Statistical Analysis

All endpoints were summarized descriptively, by randomized treatment arm, visit, and study phase. For continuous variables, change from baseline was summarized where relevant, and effect sizes (between randomized arms and within each arm over time) were reported using Hedge's *g* [95% CI]. Study phases were the RCT (first 12 weeks), OLE (second 12‐week period) for Sirona participants, or first 12 weeks of Sirona for ex‐Placebo participants. Analyses were based on actual data; missing values were treated as missing in the statistical evaluation.

Approximately 28 individuals completing the 6‐month study were planned in the Sirona arm to provide data on safety, tolerability, and feasibility and preliminary data on efficacy. Although no formal sample size calculation was performed for this feasibility study, this sample was deemed sufficient to provide data to inform the design of a larger, fully powered trial in the future. Placebo participants were included in a ratio of 1:3 Sirona participants, resulting in an overall sample size plan for 30 Sirona and 10 Placebo participants.

Three analysis populations were predefined: full analysis set/intention to treat (ITT) included all randomized participants (presented for baseline characteristics), safety analysis set included all randomized patients receiving at least one dose of study treatment (presented for safety data), and efficacy analysis set included participants with data available at baseline and the time point of interest. When conducting the preliminary analysis of the weight‐related parameters, consideration of likely inclusion of participants in an efficacy study was given (e.g., lack of substantial weight loss immediately prior to study enrollment). This resulted in an additional, post hoc, “index” analysis set: data points were removed from participants who (a) would not meet the inclusion criteria for a pivotal efficacy study and (b) were statistical outliers (> 2 SD from the sample mean). Prespecified exploratory subgroup analyses were conducted based on age, sex, diabetes status, BMI class, and compliance with dosing (descriptives and Hedge's *g* for effect size), and the relationship between baseline characteristics (age, sex, and BMI class) and %TBWL was assessed using Pearson's correlation and receiver operating characteristic (ROC) curve analysis. Statistical analyses were conducted using SAS version 9.4 and IBM SPSS version 28.

## 3 Results

### 3.1 Participants

**FIGURE 1:**
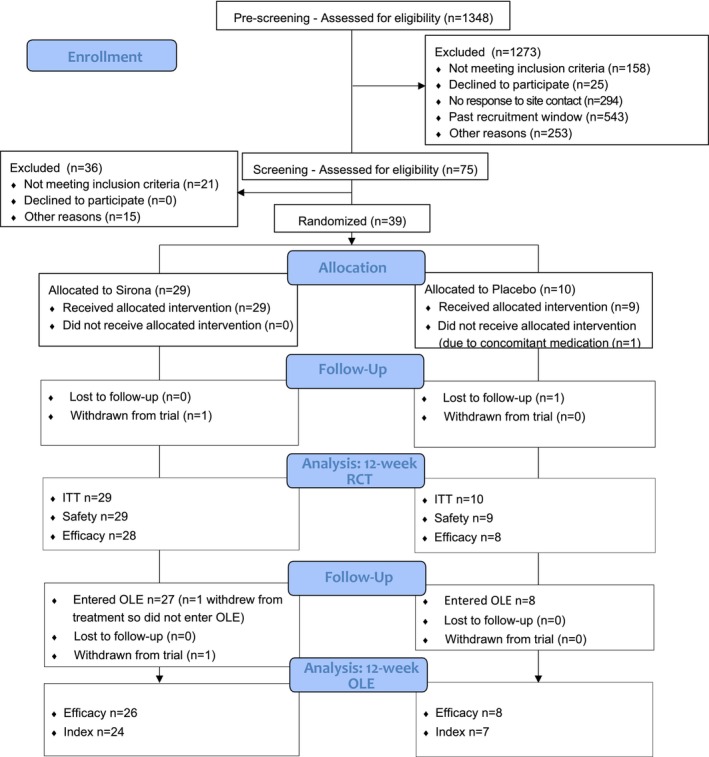
CONSORT flow diagram of participants through the study.

Of the 1348 volunteers prescreened, 75 participants were consented and screened for eligibility, then 39 participants were enrolled and randomized to either the Sirona arm (*n* = 29) or the Placebo arm (*n* = 10) (Figure 1).

Overall, participants were predominantly female (76.9%) and White British (61.5%). Participants in the Sirona arm were slightly older, with a higher percentage of females and more diverse ethnicities than those in the Placebo arm (Table 1). The overall mean BMI was 35.9 kg/m^2^, with slightly higher baseline weight and BMI in the Placebo arm; full details of baseline clinical characteristics are in Table 2 (with additional metrics reported in Table S2).

**TABLE 1:** Demographic characteristics (ITT population).

	Randomized treatment group		Total (*N* = 39)
	Placebo (*N* = 10)	Sirona (*N* = 29)	
Age (years)			
Mean (SD)	36.7 (8.07)	41.9 (8.39)	40.6 (8.52)
Median	35.5	41.0	39.0
Range	29.0, 57.0	28.0, 58.0	28.0, 58.0
*N*	10	29	39
Missing	0	0	0
Sex, *n* (%)			
Female	7 (70.0%)	23 (79.3%)	30 (76.9%)
Male	3 (30.0%)	6 (20.7%)	9 (23.1%)
Gender, *n* (%)			
Female	6 (60.0%)	23 (79.3%)	29 (74.4%)
Male	3 (30.0%)	6 (20.7%)	9 (23.1%)
Other	1 (10.0%)	0 (0.0%)	1 (2.6%)
Ethnicity, *n* (%)			
White—British	9 (90.0%)	15 (51.7%)	24 (61.5%)
White—Other background	0 (0.0%)	8 (27.6%)	8 (20.5%)
Black/Black British—Carribean	1 (10.0%)	0 (0.0%)	1 (2.6%)
Black/Black British—African	0 (0.0%)	1 (3.4%)	1 (2.6%)
Asian or Asian British—Indian	0 (0.0%)	1 (3.4%)	1 (2.6%)
Asian—Other background	0 (0.0%)	1 (3.4%)	1 (2.6%)
Chinese	0 (0.0%)	2 (6.9%)	2 (5.1%)
Other	0 (0.0%)	1 (3.4%)	1 (2.6%)
Country, *n* (%)			
UK	9 (90.0%)	18 (62.1%)	27 (69.2%)
Other	1 (10.0%)	11 (37.9%)	12 (30.8%)
Other country, *n* (%)			
Belgium—Military	0 (0.0%)	1 (9.1%)	1 (8.3%)
Bulgaria	0 (0.0%)	1 (9.1%)	1 (8.3%)
Dominican Republic	0 (0.0%)	1 (9.1%)	1 (8.3%)
Ghana	0 (0.0%)	1 (9.1%)	1 (8.3%)
Greece	0 (0.0%)	1 (9.1%)	1 (8.3%)
Northern Ireland	1 (100.0%)	0 (0.0%)	1 (8.3%)
Philippines	0 (0.0%)	1 (9.1%)	1 (8.3%)
Poland	0 (0.0%)	2 (18.2%)	2 (16.7%)
South Africa	0 (0.0%)	3 (27.3%)	3 (25.0%)

**TABLE 2:** Baseline clinical characteristics summary (ITT population).

	Randomized treatment group		Total (*N* = 39)
	Placebo (*N* = 10)	Sirona (*N* = 29)	
BMI (kg/m^2^)			
Mean (SD)	36.7 (3.14)	35.6 (3.31)	35.9 (3.26)
Range	30.4, 41.0	30.3, 41.8	30.3, 41.8
Waist circumference (cm)			
Mean (SD)	110.6 (9.95)	108.1 (10.60)	108.7 (10.37)
Range	89.0, 122.0	87.0, 132.0	87.0, 132.0
Waist to hip ratio			
Mean (SD)	0.9 (0.10)	0.9 (0.10)	0.9 (0.10)
Range	0.8, 1.1	0.7, 1.2	0.7, 1.2
Weight (kg)			
Mean (SD)	108.1 (15.09)	100.4 (11.77)	102.4 (12.95)
Range	84.9, 131.6	81.4, 122.9	81.4, 131.6
Temperature (C)			
Mean (SD)	36.7 (0.27)	36.6 (0.39)	36.6 (0.37)
Range	36.1, 37.1	36.0, 37.5	36.0, 37.5
Systolic BP (mmHg)			
Mean (SD)	124.0 (14.01)	127.6 (14.78)	126.7 (14.49)
Range	108.0, 149.0	110.0, 179.0	108.0, 179.0
Diastolic BP (mmHg)			
Mean (SD)	73.6 (9.83)	77.0 (8.08)	76.1 (8.56)
Range	62.0, 94.0	59.0, 93.0	59.0, 94.0
Heart rate (beats/min)			
Mean (SD)	67.6 (10.67)	74.8 (9.41)	73.0 (10.12)
Range	51.0, 84.0	57.0, 96.0	51.0, 96.0
Respiratory rate (breaths/min)			
Mean (SD)	15.4 (2.27)	15.8 (2.18)	15.7 (2.18)
Range	12.0, 18.0	12.0, 19.0	12.0, 19.0
Missing	0	1	1
Glucose (mmol/L)			
Mean (SD)	4.7 (0.53)	4.9 (0.56)	4.9 (0.55)
Range	4.1, 5.5	3.9, 6.0	3.9, 6.0
Missing	0	1	1
HbA1c (mmol/mol)			
Mean (SD)	33.3 (3.43)	35.3 (4.35)	34.8 (4.19)
Range	26.0, 37.0	29.0, 47.0	26.0, 47.0
Insulin (mu/L)			
Mean (SD)	9.3 (6.12)	10.6 (6.28)	10.2 (6.18)
Range	3.2, 22.3	4.2, 28.5	3.2, 28.5
Missing	0	2	2
Triglycerides (mmol/L)			
Mean (SD)	0.8 (0.23)	1.3 (0.67)	1.2 (0.63)
Range	0.5, 1.1	0.5, 3.4	0.5, 3.4
Cholesterol (mmol/L)			
Mean (SD)	4.5 (1.09)	5.3 (1.09)	5.1 (1.13)
Range	2.9, 6.5	3.8, 7.4	2.9, 7.4

### 3.2 Feasibility

Thirty‐nine participants were enrolled in the randomized treatment period (97.5% of the planned sample size). Patients were randomized at a rate of 5.4 per month over a 4‐month recruitment period. The majority of participants received treatment (one enrolled participant was not dosed due to concomitant medication at the time of first dosing), with high adherence to the agreed dosing schedule (RCT Sirona: 95.2 [11.0]%; RCT Placebo: 97.8 [3.5]%; OLE Sirona: 95.9 [7.5]%; OLE ex‐Placebo: 97.3 [6.6]%). Participants were retained in the trial up to the end of RCT (36/38; 94.7%) and the end of OLE (34/28; 89.5%), with excellent completion rates. These data support the feasibility of this therapy and confirm trial feasibility.

### 3.3 Tolerability

#### 3.3.1 Compliance With Dosing

During the RCT period, 18 participants (62.1%) in the Sirona arm and 5 participants (55.6%) in the Placebo arm took the maximum dose (two pills per day) or their agreed dose. Four participants (all in the Sirona arm) used the flexible dosing specified in the protocol and settled at one pill per day starting from Weeks 4 to 7. During the OLE period, 14 participants (53.8%) in the Sirona arm and 4 participants (50.0%) in the Placebo arm took the maximum dose (two pills per day) or their agreed dose. Accountability data were missing from one participant in the Sirona arm.

#### 3.3.2 Participant Experience Questionnaire Ratings

During the RCT period, the majority of participants found the pills to be moderately, very, or extremely tolerable to swallow (Sirona: 78.6%; Placebo: 75.0%; Table S3a), and during the OLE period, the majority were very or extremely satisfied with the number and frequency of pills (Sirona: 89.3%; Placebo: 75.0%; Table S3b).

#### 3.3.3 Ratings on MSSS

The majority of protocol‐specified daily ratings using the MSSS reported no symptoms and only one report of vomiting. The number of MSSS ratings at each scale level is reported in Table S4a. Nausea was largely reported (73.0%) during the first 3 weeks of the trial, coinciding with the initial dose‐escalation phase. A similar pattern was reported in the OLE period for the ex‐Placebo group (Table S4b).

### 3.4 Safety

During the RCT period, 31 (79.5%) participants had at least one AE, 5 (55.5%) in the Placebo group and 26 (89.7%) in the Sirona group. There were 115 AEs in total, 6 AEs in the Placebo group and 109 in the Sirona group (safety sample, Table 3). To note, every count of nausea that was reported by participants on the MSSS (score of 2 and above) was logged as an AE even if it was transient, that is, for only 30 min in duration, and successive experiences of nausea were reported as discrete AEs. AEs were primarily gastrointestinal symptoms, graded as mild, and resolved with no action. There were 26 AEs reported in the OLE period by 13 (50.0%) participants in the Sirona arm continuing treatment (Table 3); only 6 (23%) were deemed to be possibly related to Sirona. There were 30 AEs reported in OLE by 6 (75.0%) participants in the ex‐Placebo arm (Table 3), primarily graded mild (73.3%) and as gastrointestinal disorders (76.7%). This is similar to previous findings in the first 12 weeks of treatment for those in the Sirona arm. There were no serious AEs during the whole trial. Finally, the trial Sponsor Steering Committee, led by an independent bariatric surgeon, reviewed the gastroscopy, vital signs, and clinical laboratory findings and agreed that there were no concerning findings.

**TABLE 3:** AEs by severity, relatedness, action taken, outcome, and system organ class.

RCT	Randomized treatment group		Total RCT (*N* = 115)
	Placebo (*N* = 6)	Sirona (*N* = 109)	
Severity, *n* (%)			
Mild	4 (66.7%)	85 (78.0%)	89 (77.4%)
Moderate	2 (33.3%)	24 (22.0%)	26 (22.6%)
Serious, *n* (%)			
No	6 (100.0%)	109 (100.0%)	115 (100.0%)
SAR, *n* (%)			
No	6 (100.0%)	109 (100.0%)	115 (100.0%)
SUSAR, *n* (%)			
No	6 (100.0%)	109 (100.0%)	115 (100.0%)
Related to IMD, *n* (%)			
Unrelated	5 (83.3%)	23 (21.1%)	28 (24.3%)
Possible	1 (16.7%)	39 (35.8%)	40 (34.8%)
Probable	0 (0.0%)	38 (34.9%)	38 (33.0%)
Related	0 (0.0%)	9 (8.3%)	9 (7.8%)
Action taken, *n* (%)			
None	5 (83.3%)	94 (86.2%)	99 (86.1%)
Discontinued permanently	0 (0.0%)	1 (0.9%)	1 (0.9%)
Discontinued temporarily	1 (16.7%)	12 (11.0%)	13 (11.3%)
Delayed dose	0 (0.0%)	2 (1.8%)	2 (1.7%)
Special interest, *n* (%)			
Yes	2 (33.3%)	68 (62.4%)	70 (60.9%)
No	4 (66.7%)	41 (37.6%)	45 (39.1%)
Outcome, *n* (%)			
Resolved	5 (83.3%)	104 (95.4%)	109 (94.8%)
Ongoing	1 (16.7%)	2 (1.8%)	3 (2.6%)
Unknown	0 (0.0%)	3 (2.8%)	3 (2.6%)
System organ class, *n* (%)			
Gastrointestinal disorders	2 (33.3%)	90 (82.6%)	92 (80.0%)
Hepatobiliary disorders	1 (16.7%)	0 (0.0%)	1 (0.9%)
Immune system disorders	0 (0.0%)	2 (1.8%)	2 (1.7%)
Infections and infestations	0 (0.0%)	5 (4.6%)	5 (4.3%)
Injury, poisoning, and procedural complications	0 (0.0%)	2 (1.8%)	2 (1.7%)
Metabolism and nutrition disorders	0 (0.0%)	2 (1.8%)	2 (1.7%)
Musculoskeletal and connective tissue disorders	1 (16.7%)	2 (1.8%)	3 (2.6%)
Nervous system disorders	1 (16.7%)	4 (3.7%)	5 (4.3%)
Pregnancy, puerperium, and perinatal conditions	0 (0.0%)	1 (0.9%)	1 (0.9%)
Renal and urinary disorders	0 (0.0%)	1 (0.9%)	1 (0.9%)
Reproductive system and breast disorders	1 (16.7%)	0 (0.0%)	1 (0.9%)

OLE	Ex‐Placebo arm: Sirona treatment weeks 1–12	Sirona arm: Sirona treatment weeks 13–24	Total OLE: (*N* = 56)
	Total AEs = 30	Total AEs = 26	
Severity, *n* (%)			
Mild	22 (73.3%)	22 (84.6%)	44 (78.6%)
Moderate	8 (26.7%)	4 (15.4%)	12 (21.4%)
Serious, *n* (%)			
No	30 (100.0%)	26 (100.0%)	56 (100%)
SAR, *n* (%)			
No	30 (100.0%)	26 (100.0%)	56 (100%)
SUSAR, *n* (%)			
No	30 (100.0%)	26 (100.0%)	56 (100%)
Related to IMD, *n* (%)			
Unrelated	11 (36.7%)	20 (76.9%)	31 (55.4%)
Possible	10 (33.3%)	6 (23.1%)	16 (28.6%)
Probable	9 (30.0%)	0	9 (16.1%)
Action taken, *n* (%)			
None	26 (86.7%)	20 (76.9%)	46 (82.1%)
Discontinued permanently	0	1 (3.8%)	1 (1.8%)
Discontinued temporarily	4 (13.3%)	5 (19.2%)	9 (16.1%)
Special interest, *n* (%)			
Yes	19 (63.3%)	2 (7.7%)	21 (37.5%)
No	11 (36.7%)	24 (92.3%)	35 (62.5%)
Outcome, *n* (%)			
Resolved	26 (86.7%)	17 (65.4%)	43 (76.8%)
Ongoing	4 (13.3%)	9 (34.6%)	13 (23.2%)
System organ class, *n* (%)			
Blood and lymphatic system disorders	0	1 (3.8%)	1 (1.8%)
Cardiac disorders	2 (6.7%)	0	2 (3.6%)
Gastrointestinal disorders	23 (76.7%)	11 (42.3%)	34 (60.7%)
General disorders and administration site conditions	0	2 (7.7%)	2 (3.6%)
Infections and infestations	1 (3.3%)	4 (15.4%)	5 (8.9%)
Injury, poisoning, and procedural complications	1 (3.3%)	1 (3.8%)	2 (3.6%)
Musculoskeletal and connective tissue disorders	2 (6.7%)	2 (7.7%)	4 (7.1%)
Nervous system disorders	1 (3.3%)	2 (7.7%)	3 (5.4%)
Renal and urinary disorders	0	2 (7.7%)	2 (3.6%)
Skin and subcutaneous tissue disorders	0	1 (3.8%)	1 (1.8%)

Abbreviations: IMD, investigational medical device; SAR, serious adverse reaction; SUSAR, suspected unexpected serious adverse reaction.

### 3.5 Preliminary Efficacy

#### 3.5.1 Weight‐Related Parameters

**FIGURE 2:**
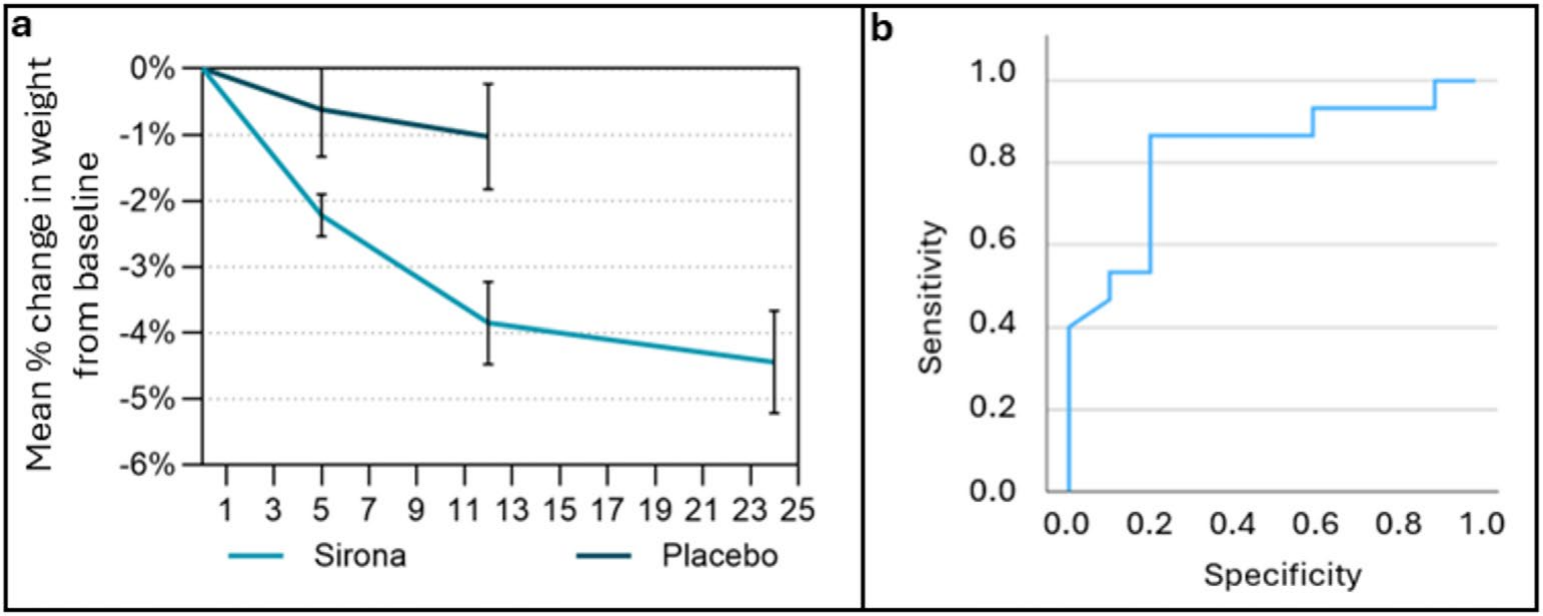
(a) Mean %TBWL from baseline over study period here and (b) ROC analysis showing the predictive ability of baseline BMI for identifying treatment nonresponse. Error bars are SEM.

Preliminary efficacy findings regarding weight loss, weight‐related parameters (index sample, Figure 1), and changes in dietary intake are presented using the index sample analysis set. A greater mean percentage change in weight was found for the Sirona arm versus the Placebo arm: −3.8% versus −1.0%, respectively (Hedge's *g* = −1.0 [−1.8, −1.0]; Figure 2). Body weight (Sirona = −3.8 kg; Placebo = −1.0 kg; Hedge's *g* = −1.0 [−1.8, −1.0]) and waist circumference (Sirona = −4.7 cm; Placebo = −0.1 cm; Hedge's *g* = −0.5 [−1.2, 0.4]) decreased in both arms over the 12‐week RCT (Table S5). After 24 weeks of Sirona, mean TBWL from baseline was 4.4% (Figure 2a), and the mean absolute change in body weight was −4.34 kg (Hedge's *g* = 1.2 [0.6, 1.7]). The maximum reported TBWL over 24 weeks was 13.5%. Changes in weight‐related parameters for the ex‐Placebo arm in the OLE period are reported in Table S5.

Although there were no clear differences in treatment response based on age or sex (Table S6), a differential treatment response was seen based on BMI class (class I = 30–34.9; class II+ > 35) following 24 weeks of Sirona treatment (class I = −6.4%; class II+ TBWL = −3.1%; Hedge's *g* = 0.9 [0.1, 1.7]). A correlation of *r* = −0.44 was found between baseline BMI and %TBWL at 24 weeks (*n* = 24), indicating that individuals with a lower baseline BMI had greater weight loss in the trial. Furthermore, a ROC curve analysis was conducted to evaluate the predictive ability of baseline BMI for identifying treatment nonresponse (defined as less than clinically meaningful weight loss of < 5%) at 24 weeks (*N* = 24). The area under the curve was 0.8, which indicates that there is an approximately 82% chance that a randomly selected nonresponder had a higher baseline BMI than a randomly selected responder (Figure 2b). Therefore, higher baseline BMI is associated with a lower likelihood of Sirona treatment response.

#### 3.5.2 Dietary Intake

**FIGURE 3:**
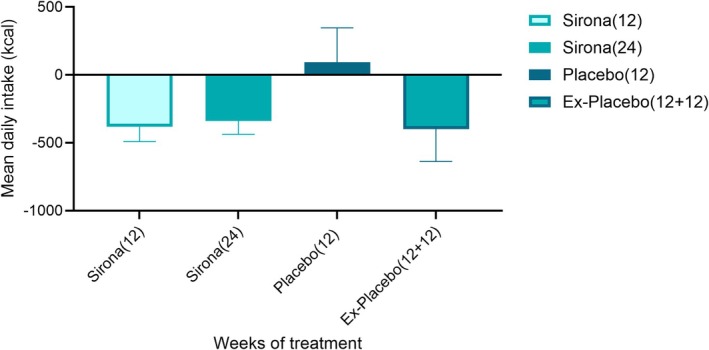
Mean change from baseline in dietary intake (kcal) in Sirona arm after 12 and 24 weeks and Placebo arm after 12 weeks of Placebo and 12 weeks of Sirona. Error bars are SEM.

Mean daily intake was reduced following 12 weeks of dosing with Sirona (−382.5 kcal, SD = 519.3 kcal, *n* = 23 versus 93.5 kcal, SD = 670.3, *n* = 7; *g* = −0.8 [−1.7, 0.0]) for Placebo (Figure 3). For participants in the Sirona arm, a reduction in mean daily intake was maintained after 24 weeks with a medium effect size (change from baseline at Week 24: mean = −338.2 kcal, SD = 486.7 kcal; *g* = 0.7 [0.2, 1.1]). Participants who crossed over to Sirona in the OLE period (ex‐Placebo) also showed a reduction in dietary intake (change from baseline mean = −398.6 kcal, SD = 631.0 kcal; *g* = 0.6 [−0.2, 1.2]).

#### 3.5.3 Metabolic Profile

Change in metabolic profile was assessed, including fasting glucose, fasting insulin, HbA1c, triglycerides, cholesterol, liver function tests (ALT, ALP), and CRP (Table S7). No clinically significant negative changes to the biomarkers, as well as specific micronutrients (vitamin B12, folate, and ferritin), were reported, indicating normal kidney, liver, and thyroid function, with no nutrient deficiency, following treatment with Sirona. Moreover, 4/7 abnormal ALT (fatty liver indicator) values at screening normalized over 12‐week Sirona treatment.

Participants with prediabetes were enrolled (*n* = 6), who had a greater scope for change with treatment. A mean reduction in HbA1c of −2.2 (2.4) mmol/mol was reported in the participants who met the criteria for prediabetes at screening. This suggests a favorable trend of an improvement in glycemia.

## 4 Discussion

The primary objectives of this trial, to confirm tolerability, safety, and feasibility, have been met. Sirona was found to be highly tolerable, as assessed through compliance with dosing, direct participant reports on the questionnaire, and infrequent scores above 2 (mild nausea) on the MSSS. Importantly, no serious AEs were reported in this trial. AEs were mild in nature, commonly gastrointestinal and without negative changes in metabolic profile, and did not impact the ability to continue with treatment. Feasibility outcomes have shown trial processes were robust, with enthusiasm for recruitment, strong adherence to measures, and compliance with treatment.

The experience of gastrointestinal side effects was expected following an initial pilot study [25] and the known gastrointestinal AEs associated with other weight loss treatments [20, 21]. Nausea was found to vary both over time within participants and between participants. Primarily, nausea was often experienced in the first 3 weeks, coinciding with the dose escalation period, rather than throughout the treatment period. The sustained weight loss observed throughout the entire trial period, extending beyond the initial 5 weeks and into the OLE period, suggests that nausea is not necessary to drive the weight loss associated with Sirona. This aligns with research demonstrating nausea is not the primary driver of weight loss with AOMs [34].

Although this trial was primarily designed to assess feasibility, safety, and tolerability, the opportunity to investigate preliminary efficacy effects in a small sample size was provided. Preliminary effects of Sirona on weight loss and reduced dietary intake were positive compared to placebo over 12 weeks, and clinical performance remained encouraging over 24 weeks of treatment, suggesting that the initial effects were maintained over the longer term. The safety, tolerability, and reduced dietary intake in the participants who crossed over to Sirona in the OLE period served to corroborate these initial findings.

Response to Sirona varied across participants within the conservative dosing protocol for this initial study. However, the variability strengthens the evidence for adaptive dosing during the initial treatment to find the optimum dose for individuals, akin to dose titration with AOMs [35]. Adaptive dose titration also allows the potential to augment the treatment response to higher doses. Moreover, the exploratory analysis of treatment responders suggested that those with a lower baseline BMI may be more likely to respond with greater weight loss to Sirona than those with a higher BMI. This is in keeping with previous research that reported those with class I obesity may have lower gastric volume and lower volume to fullness ratio [36], and this may relate to the mode of action of Sirona, likely to be through a mechanical, space‐occupying mechanism, together with downstream metabolic effects, akin to the gastric balloon [37, 38, 39]. Finally, this preliminary data suggest that treatment with Sirona may compare favorably with other treatments for weight management. Both hydrogel‐based medical devices achieved placebo‐adjusted weight loss of 2% at 6 months [20, 21]. Moreover, the STEP‐1 trial demonstrated 3.7% placebo‐adjusted TBWL at 12 weeks [13], comparable to the findings with Sirona for those with class I obesity (3.6%). The observed reduction in energy intake with Sirona is consistent with appetite suppression reported with pharmacological and device‐based obesity therapies. GLP‐1 receptor agonists, such as semaglutide, typically reduce caloric intake by 300–500 kcal/day through central appetite regulation [40], whereas another hydrogel‐based device demonstrated an average reduction of between 200 and 350 kcal/day in a randomized trial [20].

The findings should be considered in light of the limitations of the study design. Although over 1000 people completed the prescreening to participate in the trial, demonstrating great interest in the product, the sample size of this early stage pilot RCT had to remain relatively small, therefore limiting the generalizability of the findings. To prioritize data for participants taking Sirona in the pilot RCT, a 3:1 Sirona to Placebo ratio was chosen, to provide the most data possible for the Sirona arm up to 24 weeks of treatment within the study constraints. However, due to one withdrawal and one lost‐to‐follow‐up, only eight participants remained in the Placebo group, which led to high variability in many of the outcomes, making interpretation difficult. As this was primarily a feasibility trial, the duration of the RCT was kept to 12 weeks, although the inclusion of the open‐label period, while introducing known biases [41], did provide treatment data for some participants over 24 weeks, akin to trials of other medical devices for weight management [20, 21]. Although self‐reported intake data are often associated with under‐reporting [42], dietary intake was measured using a validated, online 24‐h recall measure, previously shown to be comparable to interviewer‐led data collection [33] and doubly labeled water techniques [32].

Nausea incidence was systematically assessed through daily participant‐reported MSSS questionnaires. This cumulative and aggregated proactive reporting methodology gave us a comprehensive understanding of the experience of nausea with Sirona, which has been informative to the design of future trials. However, it is likely that the method of coding every count of nausea reported as an AE through the patient‐reported nausea scale overstates real‐life experience or practice. This renders the findings less comparable with other similar trials of medical devices or medications for weight loss, such as the STEP [13] or SURMOUNT [14] series, as ongoing nausea of this kind over a week is likely only to have been counted once (as drug administered weekly). It is also likely that the patient‐reported outcome (MSSS) will have also increased awareness and therefore the incidence of nausea reporting, with daily ratings requested in the protocol.

In conclusion, these findings suggest that Sirona is safe and well tolerated, and that the product and trial procedures are feasible and acceptable to participants. Preliminary efficacy findings show reduced weight and dietary intake with Sirona treatment over 24 weeks, suggesting Sirona has potential as a nonpharmacological treatment for weight management, encompassing initial weight loss and weight loss maintenance through long‐term use, to be tested in larger trials.

## Author Contributions

James P. Byrne contributed to the conceptualization and conduct of the investigation as Chief Investigator and site PI, reviewing and editing the manuscript. Elanor C. Hinton contributed to the investigation by clinical trial management, study design, writing of the original draft, data analysis, and data interpretation. Asif Malik Humayun contributed to the data collection as site PI, including data interpretation and reviewing and editing the manuscript. Dimitri J. Pournaras contributed to the study design and data collection as site PI, data interpretation, and reviewing and editing the manuscript. Rebecca L. Elsworth contributed to the study design, data collection, data analysis, data interpretation, and reviewing and editing the manuscript. Jeffrey M. Brunstrom contributed to the study design, data interpretation, and reviewing and editing the manuscript. Julian P. Hamilton‐Shield contributed to the study design, data interpretation, and reviewing and editing the manuscript. Mark Sumeray contributed to the data interpretation and reviewing and editing the manuscript. Camilla Easter contributed to the conceptualization, study design, data interpretation, and reviewing and editing the manuscript. Hutan Ashrafian contributed to the conceptualization, study design, data interpretation, and reviewing and editing the manuscript.

## Conflicts of Interest

James P. Byrne has received institutional grant support from Oxford Medical Products Ltd. (OMP), has stock options in EMBLA, and serves as President of the British Obesity and Metabolic Specialist Society. Elanor C. Hinton has part‐time employment at both the University of Bristol and OMP. Jeffrey M. Brunstrom, Julian P. Hamilton‐Shield, and Asif Malik Humayun received institutional grant support from OMP. Dimitri J. Pournaras received institutional grant support from OMP, received consulting fees from Johnson & Johnson, Eli Lilly, Novo Nordisk, GSK, Pfizer, Medtronic, OMP, and Boston Scientific, received payment or honoraria for lectures, presentations, speakers bureaus, and manuscript writing or educational events from Johnson & Johnson, Medtronic, Novo Nordisk, Sandoz, and Eli Lilly, is Bariatric Specialty Lead for the British Obesity and Metabolic Surgery Society and Royal College of Surgeons of England, is on the steering committee for the Obesity Empowerment Network, and has stock options in Keyron. Rebecca L. Elsworth has no disclosures. Mark Sumeray has received consulting fees in the form of stock options in OMP. Camilla Easter is CEO of OMP and has stock options in OMP. Hutan Ashrafian is CMO for Harbinger Health and OMP and has stock options and received support for meeting attendance from OMP.

## Supporting information


**Data S1:** oby70066‐sup‐0001‐Supinfo.docx.

## Data Availability

The dataset will not yet be available for sharing, as it is part of ongoing regulatory submissions in the United States, United Kingdom, and European Union.
