# Supplementary material for: A Randomized, Double‐Blind, Placebo‐Controlled Pilot Trial With Open‐Label Extension of Sirona, a Hydrogel for Weight Loss
Source: Obesity (Silver Spring). 2025 Oct 15;34(1):88–100. doi: 10.1002/oby.70066 (PMC12724022; doi:10.1002/oby.70066)
Supplement: Supplementary file 1 — Data S1: oby70066‐sup‐0001‐Supinfo.docx. [file OBY-34-88-s001.docx]

**Supplementary Information for Byrne et al – Sirona Hydrogel for Weight Loss**

**Supplementary Figure S1 Trial design**


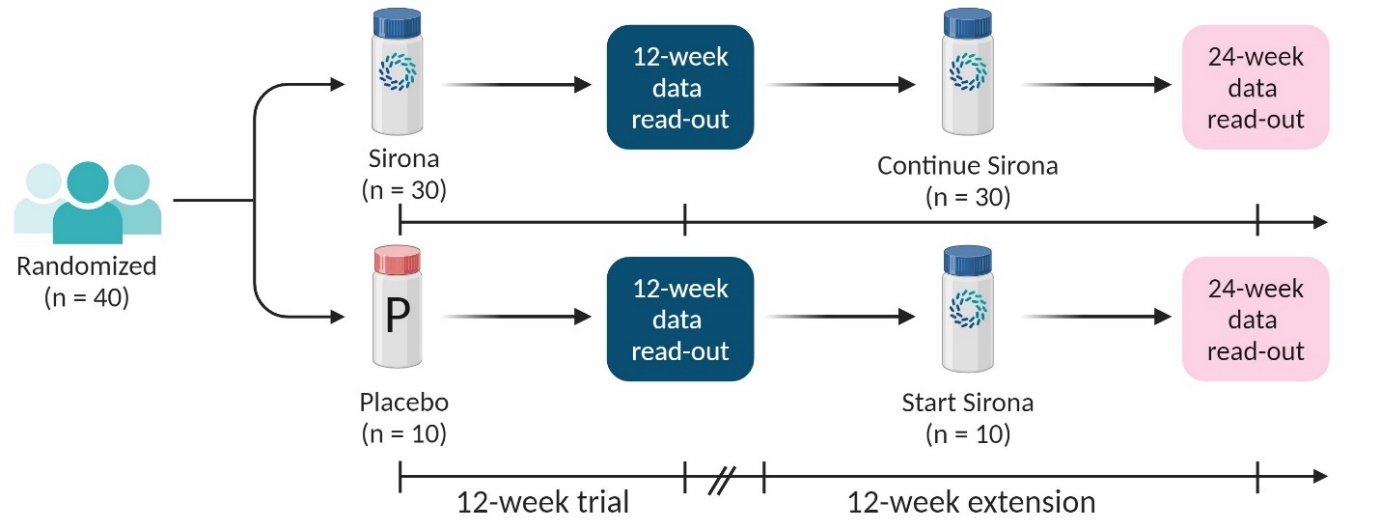


**Supplementary Table S1: Inclusion and Exclusion criteria**

| **Inclusion criteria** | **Exclusion criteria** |
| --- | --- |
| Males or females aged 18 to 65 (inclusive) years | Oral medication (excluding Omeprazole) or injectable GLP1-agonists being taken (minimum of 24 hrs washout period) |
| Healthy Volunteers | People with known human immunodeficiency virus (HIV) |
| BMI 18.5-29 for WP0 and BMI 30-40 for WP1 and WP2 | Note: Adults with HIV not on oral anti-retroviral drugs may participate in this study if their BMI is between 18.5-29 for WP0 and BMI 30-40 for WP1 and WP2 |
| Be able to understand, read and write English | Non ambulatory |
| Should not be vegan (foods used in appetite measurements will be suited to vegetarians and meat eaters) | Hiatal hernia > 3cms |
| Must be able to swallow a Sirona dummy tablet prior to enrolment on the study. Failure to swallow tablets will result in exclusion from the trial | Positive for H. pylori at screening (Participants will be allowed to continue onto the study following successful treatment) |
| Must pass the psychological evaluation (undertaken by a bariatric psychologist) | People with active gastric or duodenal ulcer disease |
| Willingness to comply with protocol requirements. | Previous gastric or oesophageal surgery |
| Signed informed consent form. | Severe oesophagitis |
|  | History of psychiatric disorders (OCD, depression, bulimia nervosa and anorexia nervosa), unless symptom-free and treatment-free for more than 2 years* |
|  | People on anticoagulant treatment or steroids |
|  | Addiction to drugs or alcohol |
|  | People with gastric or oesophageal varices |
|  | Proton pump inhibitor (PPI) current usage (Participants will be allowed to continue onto treatment following PPI prescription). |
|  | Pregnant or foreseeable pregnancy during the study or lactating females |
|  | People who smoke including cigarettes, pipes, cigars, hookahs and e-cigarettes. |
|  | People who, in the opinion of the Investigator, may be non-compliant with study schedules or procedures |
|  | People with contraindications for MRI. |
|  | People with history of stricture or regular NSAID use |
|  | People with history of abdominal adhesions |
|  | People with history of oesophageal dysmotility |
|  | Participation in another trial with an investigational device or drug |
|  | Unwilling to stay in the UK for the duration of the study |
|  | People with type one diabetes mellitus |
|  | Multiple people from the same household (residence). NB: Only one person per household can participate due to the randomisation into Sirona or Placebo study arms. |

* Potential participants who report having mental health symptoms in the past 2 years (i.e. depression symptoms on/off), who are/were not formally diagnosed or seen by a GP etc. are to be assessed by the study Psychologist.

For avoidance of doubt, people with type two diabetes may be eligible for inclusion if the following criteria are met:

1. People who are not on oral medications for their diabetes (e.g. metformin)
2. People with HbA1C in the normal range at screening

**Supplementary Table S2: Baseline clinical characteristics summary (ITT population) – additional laboratory tests (including biochemistry and micronutrients)**

|  | Randomised treatment group | |  |
| --- | --- | --- | --- |
|  | Placebo (N=10) | Sirona (N=29) | Total (N=39) |
| **LDL (mmol/L)** |  |  |  |
| Mean (SD) | 2.7 (0.97) | 3.2 (0.87) | 3.1 (0.91) |
| Range | 1.4, 4.7 | 1.9, 4.9 | 1.4, 4.9 |
| **HDL (mmol/L)** |  |  |  |
| Mean (SD) | 1.5 (0.25) | 1.4 (0.32) | 1.4 (0.30) |
| Median | 1.5 | 1.5 | 1.5 |
| Range | 1.1, 1.9 | 0.9, 2.0 | 0.9, 2.0 |
| N | 10 | 29 | 39 |
| Missing | 0 | 0 | 0 |
| **Non-HDL (mmol/L)** |  |  |  |
| Mean (SD) | 3.1 (1.02) | 3.8 (0.99) | 3.6 (1.04) |
| Median | 2.8 | 3.7 | 3.5 |
| Range | 1.7, 5.1 | 2.3, 5.8 | 1.7, 5.8 |
| N | 10 | 29 | 39 |
| Missing | 0 | 0 | 0 |
| **C-reactive protein (mg/L)** |  |  |  |
| Mean (SD) | 3.8 (3.75) | 4.3 (3.23) | 4.1 (3.33) |
| Median | 3.0 | 3.0 | 3.0 |
| Range | 1.0, 14.0 | 0.3, 12.6 | 0.3, 14.0 |
| N | 10 | 29 | 39 |
| Missing | 0 | 0 | 0 |
| **Sodium (mmol/L)** |  |  |  |
| Mean (SD) | 138.6 (2.01) | 138.9 (2.59) | 138.8 (2.43) |
| Median | 138.0 | 139.0 | 139.0 |
| Range | 136.0, 142.0 | 132.0, 143.0 | 132.0, 143.0 |
| N | 10 | 29 | 39 |
| Missing | 0 | 0 | 0 |
| **Potassium (mmol/L)** |  |  |  |
| Mean (SD) | 4.2 (0.34) | 4.2 (0.29) | 4.2 (0.30) |
| Median | 4.1 | 4.2 | 4.1 |
| Range | 3.6, 4.9 | 3.7, 4.7 | 3.6, 4.9 |
| N | 10 | 26 | 36 |
| Missing | 0 | 3 | 3 |
| **Chloride (mmol/L)** |  |  |  |
| Mean (SD) | 103.6 (2.17) | 103.4 (1.74) | 103.5 (1.83) |
| Median | 103.5 | 104.0 | 104.0 |
| Range | 100.0, 107.0 | 100.0, 107.0 | 100.0, 107.0 |
| N | 10 | 27 | 37 |
| Missing | 0 | 2 | 2 |
| **Vitamin b12 (ng/L)** |  |  |  |
| Mean (SD) | 456.5 (394.28) | 288.9 (149.38) | 331.9 (242.39) |
| Median | 307.5 | 248.0 | 254.0 |
| Range | 146.0, 1500.0 | 138.0, 770.0 | 138.0, 1500.0 |
| N | 10 | 29 | 39 |
| Missing | 0 | 0 | 0 |
| **Folate (ug/L)** |  |  |  |
| Mean (SD) | 8.2 (6.43) | 6.3 (3.48) | 6.8 (4.41) |
| Median | 5.8 | 6.0 | 6.0 |
| Range | 3.3, 23.0 | 2.7, 21.0 | 2.7, 23.0 |
| N | 10 | 29 | 39 |
| Missing | 0 | 0 | 0 |
| **Ferritin (ug/L)** |  |  |  |
| Mean (SD) | 101.7 (69.44) | 64.2 (47.14) | 73.8 (55.27) |
| Median | 80.0 | 54.0 | 57.0 |
| Range | 26.0, 224.0 | 6.0, 177.0 | 6.0, 224.0 |
| N | 10 | 29 | 39 |
| Missing | 0 | 0 | 0 |
| **T3 (pmol/L)** |  |  |  |
| Mean (SD) | 5.9 (1.24) | 5.5 (0.75) | 5.6 (0.91) |
| Median | 5.4 | 5.5 | 5.4 |
| Range | 4.8, 9.0 | 4.0, 7.0 | 4.0, 9.0 |
| N | 10 | 26 | 36 |
| Missing | 0 | 3 | 3 |
| **T4 (pmol/L)** |  |  |  |
| Mean (SD) | 11.2 (2.19) | 11.2 (1.13) | 11.2 (1.47) |
| Median | 11.3 | 11.2 | 11.2 |
| Range | 6.4, 13.9 | 8.7, 13.5 | 6.4, 13.9 |
| N | 10 | 26 | 36 |
| Missing | 0 | 3 | 3 |
| **TSH (mu/L)** |  |  |  |
| Mean (SD) | 2.1 (1.35) | 1.6 (0.47) | 1.8 (0.80) |
| Median | 1.7 | 1.5 | 1.6 |
| Range | 0.9, 5.1 | 1.0, 2.9 | 0.9, 5.1 |
| N | 10 | 29 | 39 |
| Missing | 0 | 0 | 0 |
|  | | | |

**Supplementary Table S3a: Summary of participant experience questionnaire pill and dosing schedule tolerability (Safety analysis set) at end of RCT.**

|  | | | | |
| --- | --- | --- | --- | --- |
|  | | Randomised treatment group | |  |
| Participant experience question |  | Placebo | Sirona | Total |
| How satisfactory was your experience of the number and frequency of pills? |  | (N=9) | (N=29) | (N=38) |
|  | **Response**, n (%) |  |  |  |
|  | Not at all | 0 (0.0%) | 1 (3.6%) | 1 (2.8%) |
|  | A little | 0 (0.0%) | 0 (0.0%) | 0 (0.0%) |
|  | Moderately | 2 (25.0%) | 2 (7.1%) | 4 (11.1%) |
|  | Very | 3 (37.5%) | 18 (64.3%) | 21 (58.3%) |
|  | Extremely | 3 (37.5%) | 7 (25.0%) | 10 (27.8%) |
|  | Missing | 1 | 1 | 2 |
| How tolerable did you find swallowing the pills? |  | (N=9) | (N=29) | (N=38) |
|  | **Response**, n (%) |  |  |  |
|  | Not at all | 1 (12.5%) | 3 (10.7%) | 4 (11.1%) |
|  | A little | 1 (12.5%) | 3 (10.7%) | 4 (11.1%) |
|  | Moderately | 0 (0.0%) | 12 (42.9%) | 12 (33.3%) |
|  | Very | 5 (62.5%) | 8 (28.6%) | 13 (36.1%) |
|  | Extremely | 1 (12.5%) | 2 (7.1%) | 3 (8.3%) |
|  | Missing | 1 | 1 | 2 |
|  | | | | |
| **Supplementary Table S3b: Summary of participant experience questionnaire pill and dosing schedule tolerability (Safety analysis set) at follow-up.** | | | | |
|  | | Randomised treatment group | |  |
| Participant experience question |  | Placebo | Sirona | Total |
| How satisfactory was your experience of the number and frequency of pills? |  | (N=9) | (N=29) | (N=38) |
|  | **Response**, n (%) |  |  |  |
|  | Not at all | 0 (0.0%) | 0 (0.0%) | 0 (0.0%) |
|  | A little | 1 (12.5%) | 0 (0.0%) | 1 (3.0%) |
|  | Moderately | 0 (0.0%) | 5 (20.0%) | 5 (15.2%) |
|  | Very | 4 (50.0%) | 10 (40.0%) | 14 (42.4%) |
|  | Extremely | 3 (37.5%) | 10 (40.0%) | 13 (39.4%) |
|  | Missing | 1 | 4 | 5 |
| How tolerable did you find swallowing the pills? |  | (N=9) | (N=29) | (N=38) |
|  | **Response**, n (%) |  |  |  |
|  | Not at all | 0 (0.0%) | 1 (4.0%) | 1 (3.0%) |
|  | A little | 1 (12.5%) | 2 (8.0%) | 3 (9.1%) |
|  | Moderately | 2 (25.0%) | 6 (24.0%) | 8 (24.2%) |
|  | Very | 4 (50.0%) | 13 (52.0%) | 17 (51.5%) |
|  | Extremely | 1 (12.5%) | 3 (12.0%) | 4 (12.1%) |
|  | Missing | 1 | 4 | 5 |
|  | | | | |

**Supplementary Table S4a: Number of MSSS reports during RCT**

|  | **Dose escalation** | | **Weeks 6-12** | |
| --- | --- | --- | --- | --- |
| **MSSS rating** | **Placebo: Number of patients reporting (Number of reports)** | **Sirona: Number of patients reporting (Number of reports)** | **Placebo: Number of patients reporting (Number of reports)** | **Sirona: Number of patients reporting (Number of reports)** |
| 0 - No symptoms | 9 (89) | 28 (314) | 7 (44) | 24 (169) |
| 1a - Stomach awareness | 3 (4) | 19 (41) | 3 (3) | 6 (13) |
| 1b - Abdominal discomfort | . | 10 (21) | 1 (1) | 5 (7) |
| 2 - Mild nausea | . | 7 (19) | 1 (1) | 3 (5) |
| 3 - Moderate nausea | . | 6 (6) | . | 2 (3) |
| 4 - Severe nausea | . | 1 (1) | 1 (1) | 1 (1) |
| 5 - Retching | . | 1 (1) | . | . |
| 6 - Vomiting | . | . | . | 1 (1) |

**Supplementary Table S4b: Number of MSSS reports during OLE**

| **MSSS rating** | **Ex-Placebo: Number of patients reporting** | **Ex-Placebo: Number of reports** | **Sirona: Number of patients reporting** | **Sirona: Number of reports** |
| --- | --- | --- | --- | --- |
| 0 - No symptoms | 7 | 75 | 23 | 135 |
| 1a - Stomach awareness | 2 | 2 | 4 | 4 |
| 1b - Abdominal discomfort | . | . | 4 | 4 |
| 2 - Mild nausea | . | . | 6 | 6 |
| 3 - Moderate nausea | . | . | 2 | 2 |
| 4 - Severe nausea | . | . | 1 | 1 |
| 5 - Retching | . | . | . | . |
| 6 - Vomiting | 1 | 1 | . | . |

**Supplementary Table S5: Mean (SD) percentage TBWL and change in weight, waist circumference over RCT and OLE**

| **Measure** | **Baseline** | | **End of RCT** | | **End of OLE** | |
| --- | --- | --- | --- | --- | --- | --- |
|  | **Sirona** | **Placebo** | **Sirona** | **Placebo** | **Sirona** | **Ex-Placebo** |
| TBWL (%) | na | na | 3.85 (3.01) | 1.02 (2.12) | 4.44 (3.78) | 2.33 (3.96) |
| Change in weight (kg) | 100.73 (10.85) | 106.56 (17.55) | -3.85 (2.97) | -1.04 (2.12) | -4.34 (3.60) | -2.55 (4.25) |
| Change in waist circumference (cm) | 108.8 (9.08) | 108.86 (11.19) | -4.75 (10.44) | -0.14 (5.79) | -5.17 (8.89) | -2.86 (3.99) |
| Change in BMI (kg/m^2^) | 35.73 (3.38) | 36.63 (3.54) | -1.32 (1.04) | -0.36 (0.79) | -1.52 (1.28) | -0.93 (1.59) |

**Supplementary Table S6**: **Subgroup analysis of %TBWL over 24 weeks Sirona treatment**

| **Total** | **N** | **Mean (SD) %TBWL at FU** |
| --- | --- | --- |
| **Sex** |  |  |
| Male | 5 | -5.05 (3.46) |
| Female | 19 | -4.28 (3.93) |
| **Age** |  |  |
| <40 years | 8 | -3.86 (2.00) |
| 40-54 years | 14 | -4.88 (4.75) |
| 55+ years | 2 | 3.71 (0.28) |
| **BMI Class** |  |  |
| Class I | 10 | -6.36 (3.35) |
| Class II | 14 | -3.07 (3.55) |

**Supplementary Table S7:** **Change in metabolic profile over 24 weeks Sirona treatment**

| **Biomarker** | **Mean at FU (SD)** | **Mean change from screening (SD)** | **Hedge’s g (95% CI)** |
| --- | --- | --- | --- |
| Fasting glucose (mmol/L) | 4.81 (0.43) | -0.01 (0.47) | 0.03 (-0.37, 0.42) |
| Fasting insulin (mmol/mol) | 9.15 (4.65) | -0.76 (3.46) | 0.21 (-0.20, 0.62) |
| HbA1c (mmol/mol) | 34.35 (3.96) | -0.78 (1.68) | 0.45 (0.03, 0.86) |
| Triglycerides (mmol/L) | 1.20 (0.38) | -0.11 (0.47) | 0.23 (-0.17, 0.62) |
| Cholesterol (mmol/L) | 5.18 (1.21) | -0.16 (0.65) | 0.24 (-0.16, 0.63) |
| ALT (iU/L) | 25.88 (13.65) | -1.79 (9.00) | 0.19 (-0.20, 0.58) |
| ALP (iU/L) | 79.58 (17.92) | 0.29 (10.36) | -0.3 (-0.41, 0.36) |
| CRP (mg/L) | 3.94 (3.32) | -0.31 (3.18) | 0.09 (-0.30, 0.48) |

**Supplementary Table S8: Prior and Concomitant Medications**

| **Treatment Group** | **Enrol Date** | **Drug Name** | **Indication** | **Dose** | **Units** | **Freq** | **Route** | **Start Date** | **Ongoing** | **End Date** | **ATC Code** |
| --- | --- | --- | --- | --- | --- | --- | --- | --- | --- | --- | --- |
| Placebo | 05/08/24 | Omeprazole | Duodenitist | 40 | mg | OD | PO | 05/07/24 | No | 05/28/24 | ALIMENTARY TRACT AND METABOLISM |
| Sirona | 02/27/24 | Salbutamol | Asthma | 100 | mcg | PRN | IH | 06/15/19 | Yes | . | RESPIRATORY SYSTEM |
| Sirona | 02/27/24 | Beclometasone;Formoterol | Asthma | 200 | mcg | BD | IH | 06/15/19 | Yes | . | RESPIRATORY SYSTEM |
| Placebo | 04/24/24 | Salbutamol | Asthma | 100 | mcg | PRN | IH | 11/10/15 | Yes | . | RESPIRATORY SYSTEM |
| Placebo | 04/24/24 | Beclometasone | Asthma | 200 | mcg | PRN | IH | 11/10/15 | Yes | . | RESPIRATORY SYSTEM |
| Placebo | 03/06/24 | Prochlorperazine | Migraines | 3 | mg | BD | Other | 11/13/23 | Yes | . | NERVOUS SYSTEM |
